# LDFSAM: Localization Distillation-Enhanced Feature Prompting SAM for Medical Image Segmentation

**DOI:** 10.3390/jimaging12020074

**Published:** 2026-02-10

**Authors:** Xuanbo Zhao, Cheng Wang, Huaxing Xu, Hong Zhou, Zekuan Yu, Tao Chen, Xiaoling Wei, Rongjun Zhang

**Affiliations:** 1 College of Intelligent Robotics and Advanced Manufacturing, College of Future Information Technology, College of Biomedical Engineering, Fudan University, Shanghai 200433, China; 23210860098@m.fudan.edu.cn (X.Z.);; 2 School of Computer and Information, Anhui Normal University, Wuhu 241002, China; 3 Department of Endodontics, Shanghai Stomatological Hospital, Fudan University, Shanghai 200001, China

**Keywords:** segment anything model, multi-scale feature fusion, knowledge distillation, medical image segmentation

## Abstract

Standard SAM-based approaches in medical imaging typically rely on explicit geometric prompts, such as bounding boxes or points. However, these rigid spatial constraints are often insufficient for capturing the complex, deformable boundaries of medical structures, where localization noise easily propagates into segmentation errors. To overcome this, we propose the Localization Distillation-Enhanced Feature Prompting SAM (LDFSAM), a novel framework that shifts from discrete coordinate inputs to a latent feature prompting paradigm. We employ a lightweight prompt generator, refined via Localization Distillation (LD), to inject multi-scale features into the SAM decoder as complementary Dense Feature Prompts (DFPs) and Sparse Feature Prompts (SFPs). This effectively guides segmentation without explicit box constraints. Extensive experiments on four public benchmarks (3D CBCT Tooth, ISIC 2018, MMOTU, and Kvasir-SEG) demonstrate that LDFSAM outperforms both prior SAM-based baselines and conventional networks, achieving Dice scores exceeding 0.91. Further validation on an in-house cohort demonstrates its robust generalization capabilities. Overall, our method outperforms both prior SAM-based baselines and conventional networks, with particularly strong gains in low-data regimes, providing a reliable solution for automated medical image segmentation.

## 1. Introduction

Medical image segmentation is a prerequisite for diagnosis, treatment planning, and disease monitoring across modalities such as CT, MRI, and ultrasound [1,2]. However, accurate delineation remains challenging due to low contrast, heterogeneous protocols, and the complex morphology of anatomical structures. While Convolutional Neural Networks (CNNs) like U-Net and nnU-Net, as well as recent Vision Transformers, have established the de facto standard for dense prediction, particularly semantic segmentation, their performance can degrade under domain shifts or limited annotations [3,4,5]. Moreover, traditional architectures treat segmentation as a direct supervised mapping from images to masks, in contrast to recent prompt-based segmentation paradigms that incorporate conditioning signals to enhance adaptability. As most of these medical segmentation models do not leverage such prompt-based conditioning or foundation-model priors, their generalization to new tasks remains limited.

The advent of foundation models, exemplified by the Segment Anything Model (SAM), introduces a paradigm shift towards promptable segmentation [6]. SAM uses a ViT-based image encoder, a prompt encoder and a lightweight mask decoder, and is trained on large-scale segmentation data such that it can segment arbitrary objects given flexible prompts (points, boxes, masks) in natural images. It demonstrates impressive zero-shot capabilities by segmenting arbitrary objects via flexible prompts. Motivated by this, recent works have adapted SAM for medical imaging and proposed SAM-based medical segmentation frameworks (e.g., MedSAM, SAM-Med2D) [7,8]. However, a critical limitation persists: these adaptations largely retain SAM’s reliance on explicit geometric prompts (bounding boxes or sparse points), which are inherently low-dimensional. For instance, a bounding box encodes only a coarse region of interest and carries no information about canonical object shapes, background context or appearance patterns. In medical images, where boundaries are often ambiguous and structures are deformable, rigid geometric constraints fail to capture shape nuances. More critically, box-level localization noise is easily propagated into the decoder, making segmentation accuracy heavily dependent on the precision of the prompt placement rather than the model’s semantic understanding [9].

In this work, we shift from low-dimensional geometric prompting toward feature-level conditioning within the latent space of a SAM-based medical segmentation framework. We introduce LDFSAM, which leverages distilled multi-scale representations to form two complementary latent prompts: pixel-wise Dense Feature Prompts (DFPs) to modulate spatial embeddings, and Sparse Feature Prompts (SFPs) to inject object-level semantics. These prompts are constructed by a prompt generator informed by localization-distilled detector features. Unlike standard detection that strictly regresses rigid coordinates, localization distillation allows the network to learn discrete spatial probability distributions. This probabilistic nature effectively encodes the boundary uncertainty inherent in complex medical structures. Consequently, instead of treating detection as a competing backbone, we utilize these informative features to condition the segmentation process on richer spatial and semantic cues, capturing object shape, position, and context while maintaining lightweight inference and preserving SAM’s flexible prompting interface.

We validate LDFSAM on four heterogeneous public datasets: 3D CBCT Tooth, ISIC 2018 skin lesion dermoscopy, MMOTU ovarian tumor ultrasound, and Kvasir-SEG polyp segmentation and further assess its generalization on an additional private in-house 3D CBCT tooth cohort. Consistent improvements on the in-house CBCT dataset further suggest that the latent feature prompting mechanism exhibits good generalizability from public datasets to real-world clinical scenarios.

The main contributions of this work are threefold:

1. We propose the LDFSAM, a framework that replaces low-dimensional geometric prompts with localization-aware feature prompts injected into the latent space of a SAM-based decoder. The combination of Dense Feature Prompts (DFPs) and Sparse Feature Prompts (SFPs) encodes both where the object is and what it looks like, enabling more reliable segmentation of small, crowded, and highly deformable structures.
2. A lightweight detector trained via localization distillation provides distilled multi-scale features to a dual-stream prompt encoder, improving prompt quality without increasing inference cost.
3. We conduct extensive experiments across four public benchmarks and an additional private CBCT cohort. Ablation studies show that latent feature-level prompts, comprising a pixel-wise Dense Feature Prompt (DFP) and a small set of Sparse Feature Prompts (SFPs), substantially outperform geometric box prompts, and that combining DFPs and SFPs yields consistent gains. Across different annotation budgets, LDFSAM surpasses existing SAM-based baselines and achieves Dice scores competitive with or superior to strong task-specific architectures, while maintaining modest trainable overhead and strong cross-dataset generalization.

## 2. Relative Work

### 2.1. Task-Specific Segmentation Networks

Deep learning-based medical image segmentation was first dominated by fully supervised, task-specific architectures. Fully convolutional networks (FCNs) established encoder–decoder models with skip connections as a standard solution for dense prediction [10]. Volumetric extensions and self-configuring pipelines, such as 3D U-Net, V-Net and nnU-Net, adapted this design to three-dimensional data and automatically tuned architectural and training hyperparameters for each dataset [4,11,12]. These models remain as strong baselines when sufficient pixel-wise annotations are available.

Detection-based frameworks were then adapted to support segmentation in a single network. Mask R-CNN augments a two-stage detector with a mask head and learns boxes and per-object masks jointly [13]. YOLACT and YOLACT++ factorize instance segmentation into prototype masks and per-instance coefficients and achieve real-time inference [14]. SOLOv2 formulates instance segmentation as a location-sensitive prediction problem and generates masks with dynamic kernels without explicit box regression [15]. To further capture global context and long-range dependencies, which are limited in pure CNNs, Transformer-based mechanisms have been integrated into segmentation backbones. In parallel, transformer-augmented encoder–decoder architectures TransUNet inject self-attention into U-shaped backbones to capture long-range dependencies [16], and variants such as CFATransUnet further enhance cross-scale fusion via channel-wise cross-fusion attention to narrow the semantic gap between encoder and decoder [17]. Additionally, recent studies explore adapting classification architectures for segmentation to reduce annotation dependency and computational cost [18]. Despite their efficiency and strong performance under full supervision, these models are inherently task-specific: they encode localization and segmentation priors directly into the backbone and decoder, lack a promptable interface, and do not disentangle localization cues from the segmentation latent space.

### 2.2. Segment Anything and SAM-Based Medical Segmentation

The Segment Anything Model (SAM) introduces a promptable segmentation paradigm in which a ViT-based image encoder and a prompt encoder feed a universal mask decoder trained on large-scale natural-image data [6]. SAM can segment arbitrary objects given points, boxes or masks as prompts and shows strong zero-shot performance on many non-medical tasks. On medical images, systematic studies report highly variable zero-shot performance across modalities and anatomies and strong sensitivity to prompt type, location and boundary complexity [19,20]. Moderate fine-tuning improves Dice but still struggles on small and low-contrast structures, while large-scale medical pre-training greatly improves robustness at the cost of substantial annotation and compute.

Several SAM-based medical foundation models have been proposed to address these issues. MedSAM builds a large medical image–mask corpus and trains a SAM-like model as a general medical segmenter [7]. SAM-Med2D further collects millions of 2D medical images and masks and comprehensively fine-tunes both encoder and decoder, achieving state-of-the-art performance and strong cross-dataset generalization [8]. More parameter-efficient adaptations, such as SAMed and ESP-MedSAM and DASAM [21,22,23], insert lightweight adapters, employ multi-modal distillation and design self-prompting modules to adapt SAM to multiple medical datasets with limited trainable parameters. These methods mainly modify the encoder–decoder and internal prompting of SAM. Most prompt-based approaches rely on image-space cues, such as point coordinates, scribbles, or local patches, and their dense prompts are usually obtained exclusively from the image encoder. Crucially, external localization models are not used to supply latent-space feature prompts, leaving a large portion of localization knowledge untapped.

### 2.3. From Detector-SAM Hybrids to LDFSAM

Recent work has coupled YOLOv8 detectors with SAM-family models in medical image analysis [24]. In these hybrid pipelines, YOLOv8 first localizes lesions or other regions of interest, and SAM then refines the corresponding pixel-wise masks, with applications ranging from skin cancer segmentation to multimodal ROI segmentation on ultrasound, CT and X-ray images [25,26]. These studies highlight the complementarity between fast detector-based localization and promptable segmentation.

In most such systems, however, the interface between detection and SAM is still geometric. The detector outputs boxes or coarse masks, which are converted into box prompts or similar image-space prompts for SAM, whereas the detector’s rich multi-scale features are not exploited as prompt signals. As a result, the two modules function in separate latent spaces, with limited representational interaction.

Knowledge distillation (KD) is widely used to transfer knowledge from a large teacher to a smaller student by matching softened outputs or intermediate features [27]. In object detection, KD has been extended to regression branches, but localization is often treated similarly to classification. Localization Distillation (LD) refines this idea by representing each side of a bounding box as a discrete probability distribution over spatial bins and aligning teacher–student distributions, including in a valuable localization region outside the central positive area [28]. This strategy substantially improves lightweight dense detectors by sharpening localization logits without adding inference cost. However, prior KD and LD works use the distilled detector only to obtain a better detector; the distilled representations remain confined to the detection branch. To the best of our knowledge, no existing approach repurposes localization-distilled detector features as prompts and injects them directly into the latent space of a SAM-based segmentation model. This leaves a significant opportunity to bridge detection-derived localization priors with prompt-based segmentation.

LDFSAM is situated at the intersection of these research paradigms. It uses a lightweight YOLOv8n [24] not as a final detector, but as a prompt generator whose multi-scale features are refined by localization distillation from a stronger teacher and then projected into the SAM latent space as a Dense Feature Prompt and Sparse Feature Prompts. In this way, LDFSAM redefines the prompt from low-dimensional geometry to localization-aware latent features and shifts distillation from the segmentation head to the prompt generator within a SAM-based medical segmentation framework.

## 3. Method

### 3.1. Overview of LDFSAM

Standard SAM-based approaches in medical imaging typically rely on explicit geometric prompts, such as bounding boxes or points. While effective for salient objects, these discrete coordinates essentially model the target location as a Dirac delta distribution, ignoring the inherent boundary ambiguity of complex medical structures. Specifically, the standard SAM interaction computes the segmentation mask M as $M=\mathrm{Dec}(E_{img}(I),{\mathrm{Enc}}_{prompt}(P))$, where the reliance on manual prompts P introduces labor costs and localization noise propagation.

**Figure 1:**
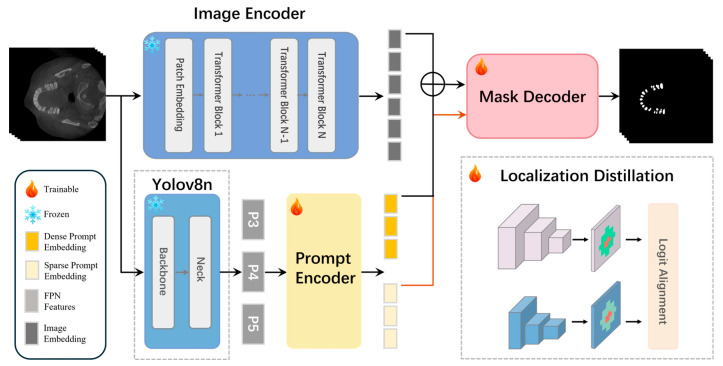
Overall architecture of LDFSAM.A frozen SAM image encoder provides image embeddings, while a lightweight YOLOv8n backbone–neck with a prompt encoder produces dense feature and sparse feature prompt embeddings that guide the SAM mask decoder. The right panel shows the training-time localization distillation from a YOLOv8x teacher to the YOLOv8n student.

To overcome these limitations, we propose LDFSAM, which shifts from discrete coordinate inputs to a latent feature-as-prompt paradigm. As illustrated in Figure 1, LDFSAM adopts a parameter-efficient fine-tuning strategy. Inspired by the SAM-Med2D paradigm, we freeze the massive SAM Image Encoder to preserve its pre-trained generalization capability while injecting lightweight adapters into the transformer blocks to capture domain-specific medical features. Simultaneously, we introduce a lightweight YOLO-based prompt generator. Instead of outputting rigid boxes, this generator extracts multi-scale features refined by Localization Distillation (LD). These refined representations are projected into complementary Dense Feature Prompt (DFP) and Sparse Feature Prompt (SFP), which collaboratively enrich the latent space with precise spatial and semantic priors. This design allows the SAM Mask Decoder to be conditioned on boundary-aware probability distributions rather than noisy coordinate values.

### 3.2. YOLO-Based Dense–Sparse Feature Prompt Encoder

We employ a lightweight YOLOv8n backbone–neck to extract multi-scale Feature Pyramid Network (FPN) features $P_{3},\;P_{4},\;P_{5}$. These features capture anatomical structures at varying resolutions, from fine-grained boundaries ($P_{3}$, 1/8 scale) to global contexts ($P_{5}$, 1/32 scale). To align these features with the SAM latent space, we propose a dual-stream projection mechanism.

#### 3.2.1. Multi-Scale Feature Fusion

First, we unify the multi-scale features into a coherent representation. To preserve high-frequency details critical for medical image segmentation, we align all feature levels to the highest-resolution scale $P_{3}$. Specifically, the coarser features ($P_{4}\;and\;P_{5}$) are bilinearly upsampled and concatenated with $P_{3}$. We then introduce a mixing $\Phi_{mix}$, composed of a 1×1 convolution for channel reduction and a 3×3 convolution for spatial smoothing, to fuse the multi-level information:

(1) $$F_{fused}=\;\Phi_{mix}\left(\mathrm{Concat}\left[P_{3},\;\mathrm{Up}\left(P_{4}\right),\;\mathrm{Up}\left(P_{5}\right)\right]\right)$$

where Up(⋅) denotes the upsampling operation and $F_{fused}\in R^{\frac{H}{S}\times \frac{W}{S}\times C}$ represents the aggregated feature map with a stride of S=8 relative to the input image.

#### 3.2.2. Dense and Sparse Feature Prompts

Dense Feature Prompt (DFP): This branch generates a pixel-wise feature map to highlight target regions. Note that the aggregated feature $F_{fused}$ retains a relatively high resolution (1/8 scale), whereas the SAM image embedding $Z_{img}$ typically operates at a lower resolution (1/16 scale). To bridge this resolution discrepancy, we employ a strided convolutional head $\Phi_{dense}$ to perform spatial downsampling while simultaneously aligning the channel dimension. The resulting dense prompt $P_{dense}$ is injected into the image embedding via a residual connection:

(2) $$Z_{cond}=Z_{img}+\alpha \cdot P_{dense}$$

where α is a learnable scaling factor initialized to 0.

Sparse Feature Prompt (SFP): Simultaneously, to abstract high-level semantic cues, we employ a Sparse Prompt Head. We apply Global Average Pooling (GAP) to squeeze the spatial information of $F_{fused}$, followed by a Multi-Layer Perceptron (MLP) to project the global vector into a high-dimensional latent space. This vector is then logically partitioned into K independent learnable tokens:

(3) $$T_{sparse}=\mathrm{MLP}\left(\mathrm{GAP}\left(F_{fused}\right)\right)$$

These tokens $T_{sparse}\in R^{K\times D}$ denote the set of K semantic tokens. By default, K=4: a sensitivity study with K∈{1,2,4,8} under identical settings shows that performance plateaus at K=4 and changes only marginally thereafter. These tokens are concatenated with the original prompt tokens, participating in the self-attention and cross-attention mechanisms of the mask decoder to provide category-specific semantic guidance.

### 3.3. Localization Distillation on YOLO Features

For efficiency, we use a lightweight detector as the latent feature proposal branch within the segmentation framework. However, such compact detectors typically exhibit reduced localization accuracy. Therefore, we adopt localization distillation from a higher-capacity YOLOv8x teacher to enhance the spatial fidelity of the features that serve as prompts.

The YOLOv8x model (~68 M parameters) is over 20× larger than the YOLOv8n model (~3 M parameters), providing substantially stronger localization distributions and richer multi-scale representations. Distilling these signals into the compact YOLOv8n network allows it to inherit the teacher’s localization sharpness while retaining the efficiency required for prompt generation within LDFSAM.

Specifically, standard bounding box regression only predicts deterministic coordinates, which often fails to capture the uncertainty of ambiguous boundaries in medical images. To address this, we employ the LD formulation. For a given bounding box edge e∈{t,b,l,r}, the network predicts continuous logits z. We first transform these logits into a probability distribution p via a Generalized Softmax with a temperature τ:

(4) $$p_{i}=S\left(z_{i},\tau \right)=\frac{e^{z_{i}/\tau }}{\sum_{j}e^{z_{j}/\tau }}$$

where τ>1 is a temperature parameter that softens the distribution to carry richer information about localization uncertainty. The student network (producing distribution $p_{S}$) learns to match the teacher’s distribution $p_{T}$ by minimizing the Localization Distillation loss $L_{LD}$, defined as the cross-entropy (or KL divergence) between them:

(5) $$L_{LD}(p_{S},p_{T})=\sum_{e\in \left\{t,b,l,r\right\}}H(p_{T}^{e},p_{S}^{e})$$

LD further enhances efficiency through Selective Region Distillation. Instead of distilling the entire feature map, we focus on strictly defined spatial subsets. The Main Distillation Region ($\Omega_{main}$) comprises standard positive samples assigned by the label assigner. To exploit informative features from ambiguous boundaries, we introduce the Valuable Localization Region (VLR) ($\Omega_{vlr}$). Let $X_{k}$ denote the maximum DIoU score for the k-th anchor. The VLR captures semi-positive anchors that provide valid localization cues but fall just below the strict positive threshold $\alpha_{pos}$:

(6) $$\Omega_{vlr}=\left\{k|{\gamma \alpha }_{pos}\leq X_{k}<\alpha_{pos}\right\}$$

where γ∈(0,1] is a hyperparameter controlling the bandwidth of this extension region. The final training objective combines the detection loss $L_{det}$ with region-weighted LD losses:

(7) $$L_{total}=L_{det}+\lambda_{main}\sum_{k\in \Omega_{main}}L_{LD}^{\left(k\right)}+\lambda_{vlr}\sum_{k\in \Omega_{vlr}}L_{LD}^{\left(k\right)}$$

The detection branch follows a YOLO-family design, using features learned during the Localization Distillation stage, while the segmentation framework extends a SAM-based promptable model augmented with our feature prompt encoder.

### 3.4. Training Strategy

The training of LDFSAM is conducted in a decoupled two-phase manner to ensure robust feature learning while maintaining computational efficiency.

Phase 1: Localization Distillation. Initially, the prompt generator branch (YOLOv8n) is pre-trained on the target dataset utilizing the LD strategy described in Section 3.3. In this phase, the high-capacity YOLOv8x teacher is kept fixed, while only the YOLOv8n student network is optimized. Upon convergence, the teacher is removed. The distilled YOLOv8n backbone–neck is then frozen to serve as a stable feature extractor, providing multi-scale FPN representations ($P_{3},P_{4},P_{5}$). These representations serve as consistent and boundary-aware prompt features for the subsequent segmentation task.

Phase 2: Segmentation Fine-tuning. Subsequently, we integrate this frozen backbone into the LDFSAM framework. To adapt the SAM backbone to medical data without catastrophic forgetting, we follow the parameter-efficient fine-tuning protocol of the SAM-Med2D paradigm. Specifically, the massive SAM image encoder remains frozen to preserve its pre-trained generalization capability, while lightweight adapter layers are injected into each Transformer block of the encoder to bridge the domain gap. During this stage, the gradients are updated only for the prompt encoder, the adapters, and the mask decoder. This strategy allows the model to learn the optimal mapping from detector features to segmentation masks with minimal trainable overhead.

## 4. Experiments

### 4.1. Datasets

To comprehensively evaluate the segmentation performance and generalization capability of LDFSAM, we utilized four diverse public datasets and one private clinical cohort covering different modalities (CBCT, Ultrasound, Dermoscopy, and Endoscopy). All datasets are split at the patient or volume level to prevent data leakage. For 3D CBCT Tooth, volumes are first divided into training and test sets, and 2D slices are extracted only after this split, ensuring that no slices from the same volume appear in both sets. For 2D datasets, splits are performed at the patient or examination level so that all frames from the same subject remain within a single split. This protocol is applied consistently across both localization distillation and segmentation training. First, the 3D CBCT Tooth dataset, derived from a large-scale dental collection, contains 129 NIFTI scans with a 0.25 mm voxel spacing, which were divided into 103 scans for training and 26 for testing following standard protocols [29]. To ensure consistency, we adopted 0.25 mm as the standard voxel spacing and performed slice extraction for all volumes. Second, we employed the ISIC 2018 benchmark for skin lesion analysis that is available; utilizing a random split of its 2594 dermoscopy images, we allocated 80% (2075 images) for training and 20% (519 images) for testing [30]. Third, for ultrasound, we utilized the OTU_2d subset of the MMOTU dataset, comprising 1469 images, which were adopted with a standard protocol of 1000 for training and 469 for testing [31]. Fourth, the Kvasir-SEG dataset, consisting of 1000 polyp images from endoscopic procedures, was randomly split into 800 images for training and 200 for testing [32]. For all random partitions, we implemented a deterministic splitting protocol using a fixed seed (2025). Specifically, sample IDs were lexicographically sorted and subjected to a single shuffle before allocating the test sets according to the predefined splits. Subsequently, 10% of the remaining training data was strictly held out as a validation set for checkpoint selection and tuning. This configuration ensured complete isolation of the test set for final performance evaluation and was uniformly applied across all comparative experiments.

In this study, we utilized an in-house dental dataset provided by Shanghai Stomatological Hospital exclusively as an external test set to evaluate model generalization. This dataset comprises 50 3D CBCT scans. Institutional permission was granted by the hospital to use their clinical 3D CBCT images for research purposes. To ensure the privacy and anonymity of the patients, their data was thoroughly anonymized during the preprocessing phase. Regarding image dimensions, the dataset had a mean axial matrix size of 565 × 565 pixels (range: 272 × 272–820 × 820), with a mean slice number of 371 (range: 92–576). In terms of physical properties, the scans maintained isotropic voxel spacing with a mean of 0.296 mm (range: 0.15–0.40) and a mean voxel volume of 0.0282 mm^3^. This corresponds to a physical field-of-view (FOV) with mean dimensions of 164.4 × 164.3 mm in the axial plane and 110.2 mm along the *z*-axis.

### 4.2. Implementation Details

We implemented the proposed LDFSAM using the PyTorch framework (version 2.3.1) on a high-performance workstation equipped with four NVIDIA RTX 3090 GPUs (24 GB VRAM each). The training was executed in the two phases described in Section 3.4.

In the Localization Distillation Stage, we utilized a high-capacity YOLOv8x as the teacher and a lightweight YOLOv8n as the student, both initialized with COCO pre-trained weights. The prompt generator was trained with an input resolution of 640×640. We employed the SGD optimizer following the standard YOLOv8 optimization recipe. Consistent with the original LD configuration, the distillation temperature τ was set to 10, and the weights for both the main and VLR distillation regions ${(\lambda }_{main},\;\lambda_{vlr})$ were set to 1.0.

For datasets that provide only segmentation masks, bounding-box annotations are generated by computing tight axis-aligned boxes $\left(x_{min},\;y_{min},x_{max},y_{max}\right)$ around each annotated instance, where $x_{min}$ and $y_{min}$ denote the minimum x- and y-coordinates of the foreground pixels, and $x_{max}$ and $y_{max}$ denote the maximum x- and y-coordinates. For images with multiple instances, one box is generated per instance. At each annotation budget, boxes are derived exclusively from the same subset of masks used for segmentation training, ensuring strict supervision parity across localization distillation and segmentation.

In the subsequent Segmentation Training Stage, image slices were resized to 256×256. We updated the prompt encoder, adapters, and mask decoder for 200 epochs using the AdamW optimizer with an initial learning rate of $1\times 10^{-4}$ and a cosine annealing schedule. The batch size was set to 16 per GPU, and the training objective was defined as the sum of Dice loss and binary cross-entropy.

### 4.3. Ablation on Dense–Sparse Feature Prompts

We first investigate the effect of using YOLO-derived features as prompts. For each dataset, we keep the SAM encoder, the mask decoder, and the localization-distilled YOLOv8 feature extractor fixed, and vary only the prompting strategy.

As shown in Table 1, using only SFP (Feature-S) yields improvements, though slightly below those of Feature-D, indicating that object-level tokens alone do not adequately capture the fine boundary-proximal details required for precise segmentation.

Table 1 compares segmentation performance across four datasets under different prompting strategies: Baseline (box), Feature-D (DFP), Feature-S (SFP), and the combined Feature-D+S. The results show consistent performance gains when using feature-based prompts, with the combined Feature-D+S achieving the highest accuracy across all datasets. Notably, despite the substantial variation in annotation scale, from the densely annotated 3D CBCT Tooth dataset to the much smaller Kvasir-SEG dataset, we can observe that the relative improvement of Feature-D+S over the Box baseline remains consistently strong. This stability suggests that the proposed dense–sparse feature prompts provide robust gains under both rich and limited supervision, indicating their effectiveness across datasets with very different annotation budgets.

### 4.4. Effect of Localization Distillation on the Prompt Generator

We investigate the impact of localization distillation on the prompt generator and its subsequent effect on segmentation performance. In this study, we keep the SAM encoder, prompt encoder, and mask decoder fixed, and only change how the YOLOv8n prompt generator is trained. Specifically, we consider four configurations: a baseline without any distillation, conventional classification logit distillation (KD), localization distillation applied only on the main positive region (LD-Main), and the full localization distillation with both main region and Valuable Localization Region (LD-Main+VLR). For each strategy, the resulting YOLOv8n is frozen and plugged into LDFSAM. The evaluation was repeated on all four datasets using different random seeds.

Table 2 reports the mean Dice scores across four datasets under four training configurations for the latent feature prompts derived from the YOLOv8n prompt generator. Without distillation (None), performance is the lowest on all datasets. Classification-logit distillation (KD) provides a clear improvement, increasing accuracy by approximately 1–2 Dice points. Applying localization distillation to the main positive region (LD-Main) further enhances segmentation performance across all datasets. The full LD scheme, which supervises both the main region and the valuable localization region (LD-Main+VLR), achieves the highest Dice scores consistently. These results indicate that richer localization supervision produces stronger feature-level prompts and leads to improved downstream segmentation performance.

**Figure 2:**
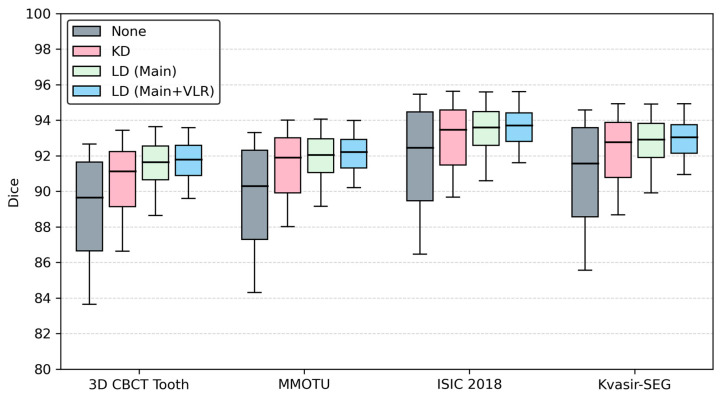
Effect of localization distillation on the prompt generator. Dice distributions of LDFSAM when the YOLOv8n prompt generator is trained with different schemes (None, KD, LD (Main), LD (Main+VLR)) on four datasets.

Figure 2 provides a more detailed view by showing the Dice distribution across individual test cases. Each box depicts the mean Dice, interquartile range (IQR), and overall spread: narrower boxes and shorter whiskers correspond to more stable segmentation behavior. Without distillation, the means are clearly lower, and the distributions are wide, indicating both reduced accuracy and larger case-to-case variance. Adding a conventional classification-logit distillation raises the mean Dice but only modestly tightens the distributions, suggesting that better class scores alone are not sufficient to produce reliable feature prompts.

In contrast, localization distillation applied to the regression branch leads to simultaneous gains in accuracy and stability. Extending LD to both the main region and the valuable localization region yields the highest mean Dice and the most compact boxes, showing that aligning teacher–student localization distributions in an extended region not only provides more informative localization cues and improves accuracy but also makes the prompt generator substantially more stable. The figure therefore shows that localization distillation can transfer most of the teacher’s localization ability into a much smaller student, so that LDFSAM benefits from strong, stable feature prompts while keeping the detector branch lightweight and efficient.

### 4.5. Comparison with Other SAM-Based Methods

We further compare LDFSAM with several SAM-based prompting methods under different annotation budgets. Since pixel-wise annotation is costly and often available only in limited quantities, an important objective is to assess the degree to which each method can generalize in low-supervision or few-shot settings. By experimenting with different proportions of annotated training masks, we systematically evaluate the data efficiency and robustness of LDFSAM relative to existing SAM-based approaches, especially when only a small fraction of training masks is available.

Table 3 details the parameter counts and computational costs of each method. Although SAM, MedSAM, and SAMed adopt lightweight parameter configurations (with approximately 4 M trainable parameters), this does not directly translate into a training speed advantage due to their resolution optimization strategies. Conversely, the models with larger parameters (SAMMed2D and LDFSAM) achieved lower GPU hours across all four datasets. LDFSAM maintains a low training time cost overhead comparable to that of SAMMed2D while effectively handling complex segmentation tasks, demonstrating its high efficiency in computational resource utilization.

To further analyze the architectural overhead, we highlight the lightweight nature of the automatic prompt generation branch. The automatic prompt generation branch operates at 640 × 640 resolution and contains approximately 3.3 M parameters with about 9.4 GFLOPs per image, running at ~122 FPS under our profiling setup. Excluding this branch, our SAM segmentation network has approximately 271.2 M parameters. Therefore, the proposed prompt branch adds only about 1.2% additional parameters relative to the segmentation model, resulting in a modest computational and memory overhead while enabling fully automatic feature-based prompting.

**Figure 3:**
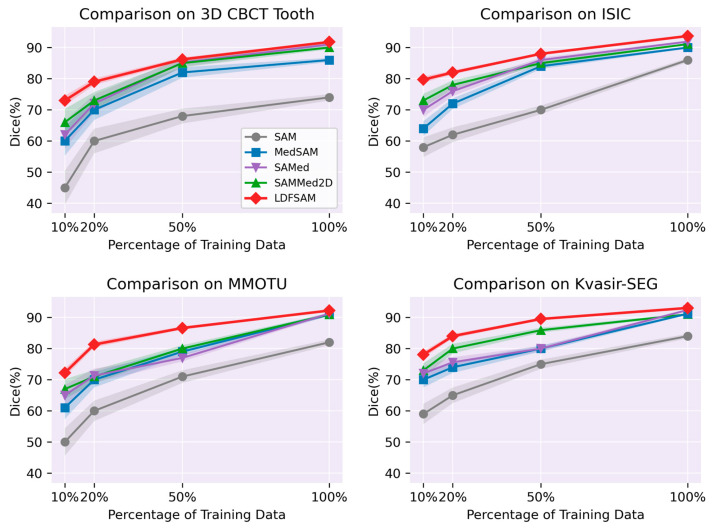
Comparison with SAM-based methods under different annotation budgets. Dice scores of SAM, MedSAM, SAMed, SAM-Med2D and LDFSAM on 3D CBCT Tooth, ISIC 2018, MMOTU, and Kvasir-SEG, using 10%, 20%, 50% and 100% of the training masks. Shaded bands represent 95% bootstrap confidence intervals over five fixed-seed runs.

Concretely, we evaluate SAM, MedSAM, SAMed and SAM-Med2D against LDFSAM on the four datasets. For each dataset and each annotation budget (10%, 20%, 50%, 100%), all stages strictly use the same subset of training images. Bounding-box annotations for localization distillation are derived exclusively from the masks within the selected subset. The YOLOv8x teacher, YOLOv8n student, and all segmentation baselines are trained on identical data partitions, ensuring strict supervision parity and fair low-label comparisons. All comparison methods were trained using their official implementations and public codebases. For LDFSAM, we followed the training protocol described in Section 4.2. The quantitative results in terms of Dice scores are presented in Figure 3.

To rigorously evaluate statistical robustness, we conducted five independent runs using fixed random seeds ({10, 42, 123, 2025, 3407}) and estimated 95% confidence intervals (CIs) via non-parametric bootstrap resampling (B = 1000). Across all datasets and all label ratios, LDFSAM consistently achieves high Dice. The advantage is particularly pronounced in the low-annotation regime (10–20%), where LDFSAM clearly outperforms competing SAM-based methods, as evidenced by the minimal overlap in their 95% confidence intervals in Figure 3. This behavior is expected: by design, LDFSAM injects strong localization priors and object-related visual cues from the LD-pretrained prompt generator through DFP/SFP. As a result, the decoder receives meaningful shape and position cues even when only a small number of pixel-wise masks are available. In other words, a substantial portion of the segmentation knowledge is carried by the feature-level prompts, which are themselves learned from detection and LD, rather than being derived solely from scarce mask supervision. As the proportion of training data increases, the performance gap to other SAM-based methods becomes smaller but remains consistent, with LDFSAM exhibiting narrower shaded areas that reflect superior stability. This demonstrates that LDFSAM offers both stronger few-shot generalization and competitive fully supervised performance.

**Figure 4:**
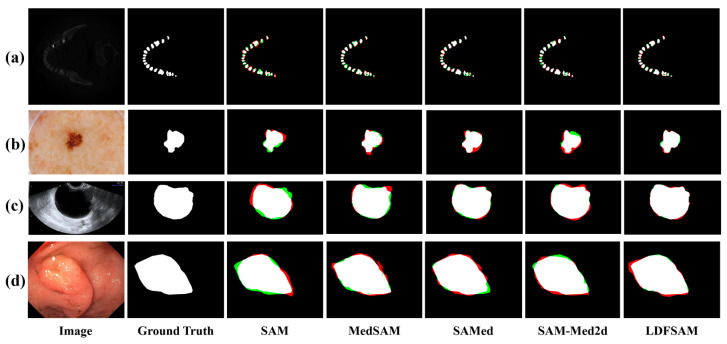
Visual comparison of segmentation results on representative cases from four datasets. (**a**) 3D CBCT Tooth; (**b**) ISIC 2018; (**c**) MMOTU; (**d**) Kvasir-SEG. From left to right: original image, ground-truth mask and predictions of different methods. White, green and red indicate correct segmentation, under-segmentation and over-segmentation, respectively.

Representative qualitative results are shown in Figure 4. Compared with other SAM-based methods LDFSAM yields masks that adhere more closely to fine tooth boundaries in CBCT slices, better capture irregular lesion shapes in dermoscopy images, and suppress spurious foreground blobs in colonoscopy scenes. These visual improvements align with the quantitative curves in Figure 3 and highlight that combining dense–sparse feature prompts with localization-distilled features leads to more accurate and stable segmentations than existing SAM-based baselines.

### 4.6. Comparison with Conventional Segmentation Networks

To place LDFSAM in the context of standard segmentation architectures, we compare it with widely used task-specific segmentation networks that do not rely on SAM or prompt engineering. We conducted paired two-sided Wilcoxon signed-rank tests on per-case Dice scores between LDFSAM and each competing method, with Holm–Bonferroni correction for multiple comparisons (α = 0.05). LDFSAM shows statistically significant improvements across all datasets, with corrected *p*-values below 0.05.

CNN-based models are initialized from random or ImageNet-pretrained weights following common practice; transformer-based models (Swin-UNet, SegFormer) use their official pretrained weights when available; nnUNet is configured with its recommended 2D setting. We tune optimizer, learning rate and data augmentation for each model based on the corresponding papers and open-source implementations, and evaluate all methods with the same Dice and IoU metrics. The quantitative results are summarized in Table 4, Table 5 and Table 6. Across all datasets, LDFSAM attains the highest Dice and IoU. On 3D CBCT Tooth, as reported in Table 4, and on MMOTU, as reported in Table 6, it slightly but consistently improves over nnUNet (e.g., Dice 91.79 vs. 91.45 on 3D CBCT Tooth; 92.21 vs. 92.03 on MMOTU), while clearly outperforming UNet, Swin-UNet, DeepLabV3+ and SegFormer by several percentage points. On ISIC 2018 and Kvasir-SEG, as shown in Table 5, the margins are larger: LDFSAM reaches 93.71 and 93.04 Dice, respectively, exceeding the best baseline (nnUNet or SegFormer) by about 1–2 points in both IoU and Dice. Overall, these tables show that a SAM-based model with a lightweight, distilled feature-prompt branch can match or surpass heavily tuned architectures such as nnUNet and modern Transformer decoders, despite relying largely on a frozen encoder and relatively modest trainable capacity.

**Figure 5:**
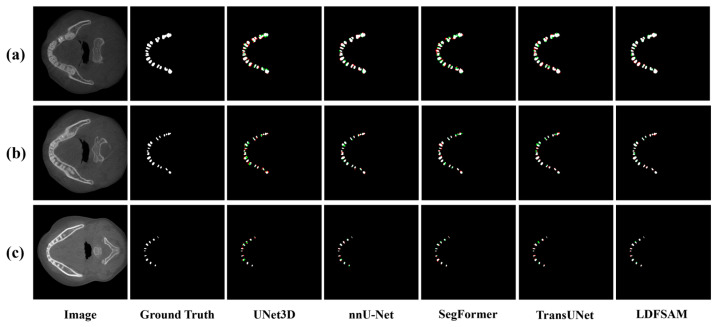
Visual comparison on 3D CBCT Tooth datasets. (**a**) Representative case 1; (**b**) Representative case 2; (**c**) Representative case 3. White, green and red indicate correct segmentation, under-segmentation and over-segmentation, respectively.

**Figure 6:**
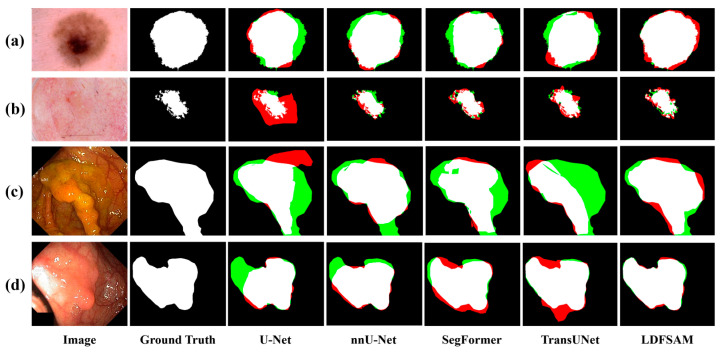
Visual comparison of segmentation results on the ISIC 2018 datasets and Kvasir-SEG datasets. (**a**) ISIC 2018, representative case 1; (**b**) ISIC 2018, representative case 2; (**c**) Kvasir-SEG, representative case 1; (**d**) Kvasir-SEG, representative case 2. White, green and red indicate correct segmentation, under-segmentation and over-segmentation, respectively.

**Figure 7:**
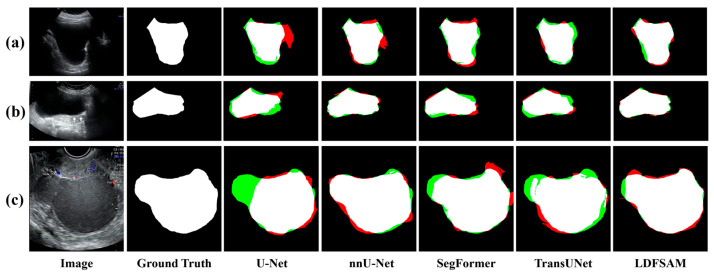
Visual comparison of ovarian tumor segmentation results on the MMOTU dataset. (**a**) Representative case 1; (**b**) Representative case 2; (**c**) Representative case 3. White, green and red indicate correct segmentation, under-segmentation and over-segmentation, respectively.

Qualitative comparisons in Figure 5, Figure 6 and Figure 7 provide further insight and complement the quantitative results. For each example, we visualize the ground-truth mask, and for every method, show an error map where red regions correspond to over-segmentation (false positives) and green regions to under-segmentation (false negatives) with respect to the ground truth. In the CBCT tooth case shown in Figure 5, several baselines either miss narrow gaps between teeth (green) or leak into the jawbone (red), whereas LDFSAM better respects the individual tooth boundaries and produces the cleanest error map. In Figure 6, which illustrates results for both skin lesions (ISIC) and colonoscopy polyps (Kvasir), CNN-based and transformer-based models exhibit irregular red or green patches along the object borders, indicating unstable delineation; conversely, LDFSAM yields tighter contours with very limited colored artifacts across both modalities. A similar trend is observed for the ovarian tumor cases (MMOTU) in Figure 7: other methods either leave residual background (green) or introduce spurious blobs (red), while LDFSAM recovers the polyp with minimal error. These visual results across all three figures are consistent with the quantitative gains in Table 1 and suggest that the dense–sparse feature prompts, enriched by localization-distilled detection features, help LDFSAM capture both global shape and fine boundary details more reliably than purely task-specific segmentation architectures.

### 4.7. Cross-Center Generalization on Privatel CBCT Data

We additionally collect a private CBCT tooth dataset from a different clinical site, containing 50 CBCT volumes with manual annotations of individual teeth. The images were pre-processed following an identical protocol to the public 3D CBCT Tooth dataset, including resampling and slice extraction.

To further examine cross-center generalization, all networks trained only on the public 3D CBCT Tooth dataset are directly applied to this private test set without any fine-tuning. As summarized in Table 7, we compute the absolute drops in IoU and Dice between the public and private CBCT results. The conventional baselines SAM-Med2D and nnUNet exhibit noticeable degradation when transferred to the private dataset, with ΔDice exceeding 1.8–2.1 percentage points, respectively. In contrast, LDFSAM shows the smallest drop (ΔIoU = 1.59%, ΔDice = 0.92%), indicating that its predictions remain much more stable under the change in scanner and patient cohort. These results suggest that the proposed dense–sparse feature prompting together with localization distillation not only improves accuracy on public benchmarks but also yields stronger cross-center generalization on real clinical CBCT data.

## 5. Discussion

### 5.1. From Geometric Prompts to Feature-Level Prompts

Standard SAM-based prompting is formulated in terms of boxes, points or scribbles. These geometric prompts are easy to interpret, but they are also extremely low-dimensional: a bounding box encodes only a coarse region of interest and carries no information about typical object shapes, background context or appearance patterns. In contrast, LDFSAM operates in the latent feature space of a SAM-based decoder. The ablation in Section 4.3 shows that introducing feature-level prompts, even those derived from a compact detector refined through localization distillation, consistently guides the decoder toward more accurate and stable segmentation outputs.

The Dense Feature Prompt (DFP) can be viewed as a high-resolution, data-driven refinement of the box within the latent space. Instead of four coordinates in image space, the decoder receives a spatial feature map that has already been aligned to the SAM image embeddings and highlights tooth or lesion regions while suppressing background responses. The Sparse Feature Prompts (SFPs) act as compact latent tokens carrying object-level cues, such as canonical shapes or coarse positions that cannot be effectively represented by a handful of points. Empirically, DFP alone clearly outperforms the Box baseline, and DFP+SFP consistently yields the best Dice on all datasets. This suggests that, for medical segmentation, the most effective “prompt” is not a purely geometric signal but a latent representation that jointly embeds localization and appearance information. In this sense, LDFSAM reframes “prompt” from drawing coordinates in image space to injecting features distilled from a learned localization model directly into the SAM latent prompt space.

### 5.2. Localization Distillation

We adopt YOLOv8 as the localization backbone for feature prompt generation due to its mature architecture, stable multi-scale feature representations, and favorable accuracy–efficiency trade-off. As our framework relies on intermediate localization features rather than final detection outputs, YOLOv8 provides a reliable and well-characterized backbone that is well suited for localization distillation and prompt generation. We note that LDFSAM is modular and can incorporate newer localization backbones as they become sufficiently stable and validated.

Building on this architectural foundation, a central element of LDFSAM is the application of localization distillation (LD) to specifically optimize the prompt generator. In our framework, the YOLOv8n branch does not serve as an independent detector; its primary role is to produce multi-scale features from which DFP and SFP are derived. As demonstrated in Section 4.4, the training strategy for this branch has a direct and substantial impact on the resulting segmentation accuracy. In particular, LD provides sharper and more reliable localization cues, which translate into more informative feature-level prompts and, consequently, stronger overall segmentation performance.

Training YOLOv8n only with the standard detection loss leads to relatively noisy localization signals, which is reflected in lower means and higher variance of Dice. Adding conventional logit distillation on the classification branch slightly improves the results, but the effect remains limited, implying that better class scores alone are not sufficient to produce high-quality latent prompts. In contrast, applying LD on the regression distributions, first on the main positive region and then on both the main region and the valuable localization region, consistently raises the Dice distributions and reduces variability. This supports the view that aligning teacher–student localization distributions is an effective mechanism to refine the geometry and sharpness of detection features, and that such refined features make more reliable dense and sparse prompts for the SAM decoder. In other words, LD is not merely an auxiliary improvement to detection; within LDFSAM, it serves as a principled mechanism for upgrading the prompt generator itself.

### 5.3. Data Efficiency and Cross-Domain Generalization

For 3D CBCT data, we process volumes via 2D slicing to remain compatible with existing SAM-based architectures and to maintain data and computational efficiency. We acknowledge that this strategy ignores explicit inter-slice correlations along the *z*-axis, which is a limitation compared with fully volumetric models. Nevertheless, operating in 2D enables a unified framework across both 2D and 3D modalities and allows effective reuse of strong 2D foundation model priors. Importantly, the proposed dense–sparse feature prompting mechanism is not inherently limited to 2D and could be extended to future 3D SAM-style models by incorporating volumetric adapters and 3D localization-based feature prompts.

Specifically, for the current 2D-based framework, this ability to effectively leverage foundation model priors translates directly into high data efficiency, as clearly demonstrated by the experiments under different annotation ratios in Section 4.5. When only 10–20% of the training masks are available, LDFSAM already achieves Dice scores that match or surpass those of SAM-based baselines trained with substantially more labels. This behavior reflects the intentional design of the framework, in which much of the task-relevant information is supplied through feature-level prompts. A significant fraction of task-relevant information is injected via the LD-pretrained prompt generator and materialized as DFP/SFP in the SAM latent space, so the segmentation branch does not need to infer all anatomical structures solely from sparse pixel-wise supervision. As the annotation volume increases, all methods see improved performance; however, LDFSAM consistently remains the top-performing approach across datasets. This indicates that the proposed feature-level prompting mechanism scales effectively from low-data to high-data regimes and provides benefits beyond those achievable through additional mask annotations alone.

The comparison with conventional segmentation networks in Section 4.5 provides a complementary perspective. Despite relying primarily on a frozen SAM encoder and a lightweight prompt encoder, LDFSAM matches or exceeds established architectures such as nnUNet, DeepLabV3+ and SegFormer on four heterogeneous public datasets. Additional experiments on a private in-house CBCT tooth dataset exhibit similar trends, suggesting that the proposed latent feature prompting strategy generalizes beyond curated benchmarks to real-world clinical data. Taken together, these results indicate that, given a strong pre-trained encoder, improving how prior knowledge is conveyed through latent-space feature prompts can be at least as important as further increasing backbone complexity. From this standpoint, LDFSAM is not merely a variant of SAM; it illustrates how detection-derived structure and principled prompt design can narrow the gap between generic foundation models and carefully engineered, task-specific segmentation networks while preserving cross-dataset generalization.

## 6. Conclusions

In this work, we proposed LDFSAM, a framework that enhances SAM-based medical segmentation by replacing geometric prompts with localization-distilled feature prompts delivered directly in the latent space. A lightweight detector, refined through localization distillation (LD), which supplies multi-scale features that are transformed into Dense Feature Prompt (DFP) and Sparse Feature Prompt (SFP), giving the decoder richer spatial and appearance cues than traditional box or point prompts.

Extensive experiments on four heterogeneous public datasets (3D CBCT Tooth, ISIC 2018, MMOTU, and Kvasir-SEG) demonstrate the effectiveness of this design. Ablation studies show that feature-based latent prompts substantially outperform box prompts, and that combining DFP and SFP yields consistent gains over using either alone. The LD study confirms that refining the prompt generator via localization-aware distillation is crucial for stable and accurate segmentation. Under varying annotation budgets, LDFSAM consistently surpasses existing SAM-based methods, especially in low-label regimes, and it achieves Dice scores that are competitive with or superior to strong task-specific segmentation networks. Additional experiments on a private in-house CBCT dataset exhibit similar improvements, indicating that the proposed latent feature prompting mechanism maintains good generalization beyond public benchmarks.

LDFSAM illustrates that carefully designed feature-level prompts in the latent space, distilled from a localization model, can significantly narrow the gap between generic foundation models and specialized medical segmentation architectures, while keeping inference light weight and modular.

## Figures and Tables

**Table 1:** Ablation experiments of bounding-box baseline and dense–sparse feature prompts validity with YOLOv8n/x backbones on 3D CBCT Tooth, ISIC 2018, MMOTU, and Kvasir-SEG datasets. ↑ denotes higher is better. Bold values indicate the best results.

Backbone	Method	3D CBCT Tooth		ISIC 2018		MMOTU		Kvasir-SEG	
		IoU (%) ↑	Dice (%) ↑	IoU (%) ↑	Dice (%) ↑	IoU (%) ↑	Dice(%) ↑	IoU (%) ↑	Dice (%) ↑
YOLOv8n	Baseline	78.91	87.36	83.55	90.28	79.34	87.88	81.67	89.54
	Feature-D	81.05	88.92	85.66	91.83	82.29	89.72	85.01	91.06
	Feature-S	79.36	88.14	84.59	91.09	81.05	88.95	84.04	90.82
	Feature-S+D	**81.89**	**89.65**	**86.65**	**92.46**	**83.13**	**90.30**	**85.16**	**91.57**
YOLOv8x	Baseline	81.64	89.30	85.62	91.86	83.01	90.23	84.50	91.10
	Feature-D	84.78	91.05	87.90	93.26	86.15	92.21	87.44	92.81
	Feature-S	83.56	90.17	86.76	92.62	84.72	91.39	86.28	92.33
	Feature-S+D	**85.98**	**91.93**	**89.05**	**93.95**	**87.11**	**92.76**	**87.62**	**93.12**

**Table 2:** Ablation experiments of distillation schemes (w/o distillation, KD distillation, LD (Main) distillation, LD (Main+VLR) distillation) validity on 3D CBCT Tooth, ISIC 2018, MMOTU, and Kvasir-SEG datasets. ↑ denotes higher is better. Bold values indicate the best results.

Method	3D CBCT Tooth		ISIC 2018		MMOTU		Kvasir-SEG	
	IoU (%) ↑	Dice (%) ↑	IoU (%) ↑	Dice (%) ↑	IoU (%) ↑	Dice (%) ↑	IoU (%) ↑	Dice (%) ↑
w/o distillation	82.03	89.65	86.94	92.46	83.16	90.30	85.53	91.57
KD distillation	85.25	91.13	88.65	93.47	86.04	91.91	87.42	92.77
LD (Main) distillation	85.69	91.64	88.98	93.59	86.10	92.05	87.82	92.91
LD (Main+VLR) distillation	**85.81**	**91.79**	**89.27**	**93.71**	**86.56**	**92.21**	**88.10**	**93.04**

**Table 3:** Comparison of model parameters and computational costs on four datasets.

Method	All	Trainable	GPU-Hours@100% Masks			
	Params (M)	Params (M)	CBCT	ISIC2018	MMOTU	Kvasir-SEG
SAM [6]	93.7	4.1	421.5	38.6	22.3	19.7
MedSAM [7]	93.7	4.1	410.4	30.8	17.2	15.3
SAMed [21]	93.9	4.2	216.7	11.9	6.4	6.0
SAMMed2D [8]	271.2	186.8	132.0	7.9	3.8	3.0
LDFSAM	274.5	190.1	134.1	8.1	3.9	3.2

**Table 4:** Comparison of segmentation performance on the 3D CBCT Tooth dataset. Bold values indicate the best results. ↑ denotes higher is better, and ↓ denotes lower is better.

Method	IoU (%) ↑	Dice (%) ↑	HD (mm) ↓	ASSD (mm) ↓	SO (%) ↑
UNet3D [11]	68.00	79.52	113.78	25.50	67.09
DenseVNet [33]	84.57	91.15	8.21	1.14	94.88
AttentionUNet3D [34]	52.52	64.08	147.10	61.10	42.49
UNETR [35]	74.30	81.84	107.89	17.95	73.14
SwinUNETR [36]	83.10	89.74	82.71	7.50	86.80
nnFormer [37]	83.54	90.66	51.28	5.08	90.89
3D UX-Net [38]	75.40	84.89	108.52	19.69	73.48
nnU-Net [4]	85.33	91.50	7.87	0.96	95.05
SegFormer [39]	85.06	91.37	9.54	1.22	93.47
TransUNet [16]	84.65	90.69	12.30	2.65	91.26
LDFSAM	**85.81**	**91.79**	**5.05**	**0.63**	**95.82**

**Table 5:** Comparison of segmentation performance on ISIC 2018 and Kvasir-SEG datasets. ↑ denotes higher is better. Bold values indicate the best results for each metric.

Method	ISIC 2018			Kvasir-SEG		
	IoU (%) ↑	Dice (%) ↑	ACC (%) ↑	IoU (%) ↑	Dice (%) ↑	ACC (%) ↑
U-Net [3]	76.77	86.55	95.00	73.04	84.56	95.50
AttU-Net [34]	78.19	87.54	95.33	75.67	86.20	95.90
CA-Net [40]	68.82	80.96	92.96	71.48	83.29	94.98
CE-Net [41]	78.05	87.47	95.40	71.98	83.72	94.91
CPF-Net [42]	78.47	87.70	95.52	71.11	83.54	94.85
CKDNet [43]	77.89	87.35	95.27	70.23	82.74	94.60
nnU-Net [4]	80.10	88.42	95.88	87.15	92.90	98.11
SegFormer [39]	87.33	92.60	97.51	86.87	92.11	97.07
TransUNet [16]	82.45	89.53	96.39	77.65	86.82	96.04
LDFSAM	**88.47**	**93.71**	**97.86**	**87.22**	**93.04**	**98.39**

**Table 6:** Comparison of segmentation performance on the MMOTU dataset. Bold values indicate the best results. ↑ denotes higher is better, and ↓ denotes lower is better.

Method	IoU (%) ↑	Dice (%) ↑	HD (mm) ↓	ASSD (mm) ↓	ACC (%) ↑
U-Net [3]	80.06	88.38	18.59	3.57	96.01
nnU-Net [4]	84.66	91.02	13.78	1.87	96.55
SegFormer [39]	82.52	90.14	15.11	2.42	96.30
TransUNet [16]	81.35	89.30	15.69	2.78	96.13
LDFSAM	**86.56**	**92.21**	**12.05**	**1.80**	**97.10**

**Table 7:** Results on the private CBCT dataset. Performance drops in IoU and Dice when transferring models from the public 3D CBCT Tooth dataset to the private CBCT dataset. ↑ denotes higher is better, and ↓ denotes lower is better. Bold values indicate the best results.

	Public Dataset		Private Dataset	
	IoU (%) ↑	Dice (%) ↑	IoU (%) ↑	Dice (%) ↑
SAM-Med2D [8]	82.36	90.10	79.71 (↓2.65)	88.24 (↓1.86)
nnUNet [4]	85.28	91.45	81.87 (↓3.41)	89.33 (↓2.12)
LDFSAM	**85.81**	**91.79**	**84.22 (↓1.59)**	**90.87 (↓0.92)**

## Data Availability

The original data presented in the study are openly available in 3D CBCT Tooth at https://github.com/ErdanC/Tooth-and-alveolar-bone-segmentation-from-CBCT (accessed on 19 December 2025), in ISIC 2018 skin lesion dermoscopy at https://challenge2018.isic-archive.com (accessed on 19 December 2025), in MMOTU ovarian tumor ultrasound at https://doi.org/10.6084/m9.figshare.25058690 (accessed on 19 December 2025), and in Kvasir-SEG polyp segmentation at https://datasets.simula.no/kvasir-seg (accessed on 19 December 2025). The in-house clinical dataset presented in this study is available from the corresponding author upon reasonable request and institutional approval, due to privacy and ethical restrictions.
